# Metanephrine and normetanephrine associated with subclinical myocardial injuries in pheochromocytoma and paraganglioma

**DOI:** 10.3389/fonc.2022.1024342

**Published:** 2022-09-27

**Authors:** Yang Yu, Chuyun Chen, Wencong Han, Yan Zhang, Zheng Zhang, Ying Yang

**Affiliations:** ^1^ Department of Cardiology, Peking University First Hospital, Beijing, China; ^2^ Echocardiography Core Lab, Institute of Cardiovascular Disease, Peking University First Hospital, Beijing, China; ^3^ Department of Urology, Peking University First Hospital, Beijing, China; ^4^ Institute of Cardiovascular Disease, Peking University First Hospital, Beijing, China; ^5^ Institute of Urology, Peking University, Beijing, China; ^6^ National Urological Cancer Center, Peking University First Hospital, Beijing, China

**Keywords:** pheochromocytoma, metanephrine and normetanephrine, subclinical myocardial injury, left ventricular global longitudinal strain, retrospective study

## Abstract

**Objective:**

To analyze the correlation between metanephrine and normetanephrines (MNs) and subclinical myocardial injuries (SMI) diagnosed by low left ventricular global longitudinal strain (LV GLS) in patients with pheochromocytoma and paraganglioma (PPGL).

**Methods:**

Seventy-six patients who underwent surgery for pheochromocytoma or paraganglioma from September 2017 to April 2022 were examined. All the patients enrolled had normal left ventricular ejection fraction (LVEF) and myocardial injury biomarkers including cardiac troponin I and B-natriuretic peptide. Univariate analysis and multivariate analysis were performed to evaluate the association of MNs and subclinical myocardial injury (SMI)(defined as LV GLS<18).

**Results:**

LV GLS of 13(17.11%) PPGL patients was less than 18. The percentage of patients with elevation of single hormone (metanephrine, normetanephrine, 3-methoxytyramine) or any one of MNs was not significantly correlated with SMI (P=0.987, 0.666, 0.128 and 0.918, respectively). All MNs elevation was associated with SMI (OR: 11.27; 95% CI, 0.94—135.24; P= 0.056). After adjusting for age, All MNs elevation was significantly correlated with SMI (OR: 16.54; 95% CI, 1.22—223.62; P= 0.035).

**Conclusion:**

MNs might be an important factor influencing myocardial function. All MNs elevation might indicate SMI. If all MNs elevated, LV GLS measurement was recommended for PPGL patients to detect SMI in the absence of decrease LVEF or other heart disease in clinical practice.

## Introduction

Pheochromocytomas and paragangliomas (PPGL) are catecholamine-producing neuroendocrine tumor and surgery is the only curative therapy at present. One of the main causes of perioperative complications and death in PPGL patients is myocardial injury, including stress cardiomyopathy, hypertrophic myocardiopathy, dilated cardiomyopathy and myocardial ischemia (1). Therefore, how to identify the myocardial injury early and implement efficacious treatments before surgery are key steps to ensure the safety during the surgery (2). In previous studies, subclinical myocardial injury (SMI) is defined as left ventricular ejection fraction (LVEF) impairment and/or elevation of myocardial injury markers, without any symptoms. This definition emphasizes that the PPGL patients do not show any clinical symptoms. Given that routine examinations before surgery consist of echocardiography and myocardial enzyme test, so called SMI is easily identifiable and attached more attention by the surgeons. Therefore, the patients with PPGL who has normal LVEF, cardiac enzyme, B-natriuretic peptide (BNP) and no symptoms seems more likely to be overlooked in clinical practice, while the cardiac function of these patients seem to be normal, but serious in fact. Recently, left ventricular global longitudinal strain (LV GLS) is given more and more attention and recognized as an accurate and sensitive index in early detection of myocardial injury before LVEF impairment (3). In this retrospective study, SMI was innovatively defined as the patients with normal LVEF, cardiac enzyme, BNP and no symptoms, but with low LV GLS. Early identification and treatment for real SMI would be helpful for perioperation management. Considering preoperative LV GLS measurement is uncommon now and lacking of studies in PPGL, complete echocardiography images before surgery are remeasured and analyzed for LV GLS in this study. Our study aims at examining the correlation between clinical parameters, especially MNs, and SMI with innovative definition in PPGL patients.

## Methods

### Study design and patient enrollment

Patients who underwent surgery for PPGL from September 2017 to April 2022 in Peking University First Hospital and tested for plasma MNs (including metanephrine (MN), normetanephrine (NMN), 3-methoxytyramine (3-MT)) before surgery were initially enrolled as potential participants (N=262). A retrospective study design was used. The patients who met any of the following criteria were excluded (1): surgery involving non-tumor organs (2); incomplete data abstracted from the electronic medical records (3); missing echocardiography images before surgery or poor image quality (unable to obtain adequate tracking quality in more than two left ventricular (LV) segments during offline strain analysis); (4) complicated with heart disease including catecholamine cardiomyopathy (CC) or LVEF <50%; (5) elevation of cardiac troponin I or hypersensitive troponin I or BNP before surgery. Finally, 76 patients were enrolled. The study protocol was approved by the ethics committee of Peking University First Hospital. Written informed consent for participation was not required and the study was conducted in accordance with the Declaration of Helsinki.

For each patient, we reviewed the medical records to collect information including patient demographics, medical history, the highest systolic blood pressure (SBP) and diastolic blood pressure (DBP) before surgery, biochemical test, and tumor size in postoperative pathologic examination. All biochemical test values were defined to be positive if beyond the upper limit of the normal range. Patients were divided into two groups: no myocardial injury (defined as LV GLS≥18) and SMI (defined as LV GLS<18) (4).

### Echocardiography exam

All patients’ echocardiographs before surgery were digitally recorded and remeasured for LV function following the echocardiography core laboratory protocol based on American Society of Echocardiography guidelines (3), with standard commercially available cardiac ultrasonography equipment of several manufacturers. LV GLS was measured offline using exported raw DICOM images and a post processing analysis package (Image-Arena, TOMTEC). Peak segmental strain values from apical 2-, 3-, and 4-chamber views were averaged to calculate peak LV GLS.

15 randomly selected patients’ LV GLS were remeasured by the same observer blinded to previous measurements with 1 week interval, and by a second observer blinded to the first one’s results to assess intra- and inter- observer variability.

### Statistical analyses

Continuous variables were presented as mean ± standard deviation or median [Percentile 25, Percentile 75]. Categorical variables were presented as number with percentage(%). Differences between the two groups were compared using Student’s t-test or the χ^2^ test. Univariate analysis and multivariate analysis were performed to evaluate the association of MNs and LV GLS. Covariates were included as potential confounders in the adjusted model if P ≤ 0.1. All P values were two-sided and were considered statistically significant if less than 0.05. All analyses were performed with statistical software (Empower(R), www.empowerstats.com; X&Y solutions, Inc., Boston, MA, USA; R [http://www.R-project.org].v4.2.0).

## Results

### Patient characteristics

In this retrospective study, the average age was 46.34 ± 15.04 years and 34 (44.74%) of the patients were male. LV GLS in 13(17.11%) patients was less than 18. Additionally, 39 (51.32%) had history of hypertension, 18 (23.68%) had history of diabetes, and 4 (5.33%) had history of stroke. LV GLS in two group were 21.94 ± 2.45 (no myocardial injury), 14.90 ± 1.81 (SMI), respectively. Elevation of single hormone (MN, NMN, 3-MT) was no different in the groups (P=0.987, 0.665 and 0.111, respectively). However, the rate of elevation of all MNs was 1.59% and 15.38% (P=0.020). And there were no differences in other factors among the groups (**Table 1**).

**Table 1:** Characteristics of all PCGL patients.

Variables	Total patients (n=76)	No myocardial injury (n=63)	Subclinical myocardial injury (n=13)	P value
Age, years	46.34 ± 15.04	47.65 ± 14.81	40.00 ± 15.13	0.095
Sex, Male, n (%)	34 (44.74)	29 (46.03)	5 (38.46)	0.617
BMI, kg/m^2^	23.58 ± 3.33	23.61 ± 3.48	23.43 ± 2.56	0.866
Smoke, n (%)	12 (15.79)	9 (14.29)	3 (23.08)	0.429
Drink, n (%)	12 (16.00)	11 (17.46)	1 (8.33)	0.429
SBPhighest before surgery, mmHg	145.38 ± 38.16	145.78 ± 40.01	143.46 ± 28.67	0.844
DBPhighest before surgery, mmHg	85.45 ± 19.73	84.65 ± 19.91	89.31 ± 19.13	0.442
Hypertension, n (%)	39 (51.32)	31 (49.21)	8 (61.54)	0.418
Stroke, n (%)	4 (5.33)	3 (4.84)	1 (7.69)	0.677
Diabetes, n (%)	18 (23.68)	14 (22.22)	4 (30.77)	0.509
elevation of hormone, n (%)				
MN	29 (38.67)	24 (38.71)	5 (38.46)	0.987
NMN	61 (80.26)	50 (79.37)	11 (84.62)	0.665
3-MT	8 (10.67)	5 (8.06)	3 (23.08)	0.111
any one of MNs	65 (85.53)	54 (85.71)	11 (84.62)	0.918
all of MNs	3 (3.95)	1 (1.59)	2 (15.38)	0.020
Tumor				
ectopic tumor, n (%)	15 (19.74)	13 (20.63)	2 (15.38)	0.665
tumor size, cm	5 (3.50-7.00)	5.00 (3.50-7.00)	5.50 (3.00-6.50)	0.291
Echocardiography				
LV GLS	20.73 ± 3.55	21.94 ± 2.45	14.90 ± 1.81	<0.001

BMI, body mass index; SBP, systolic blood pressure; DBP, diastolic blood pressure; MN, metanephrine; NMN, normetanephrine; 3-MT, 3-methoxytyramine; MNs, metanephrine and normetanephrines; LV GLS, left ventricular global longitudinal strain.

### Association between MNs and LV GLS

Univariate correlations of LV GLS included demographic parameters (age, sex, body mass index, smoke, drink, SBP highest before surgery, DBP highest before surgery, HT, stroke and Diabetes), elevation of hormone (MN, NMN, 3-MT, any one of MNs, all of MNs) and tumor condition (tumor size and ectopic tumor) (**Table 2**).

**Table 2:** Results of univariate analysis.

Variables	OR (95% CI)	P value
Age, years	0.97 (0.93, 1.01)	**0.100**
Sex, Male	0.73 (0.22, 2.49)	0.618
BMI	0.98 (0.82, 1.18)	0.864
Smoke	1.80 (0.41, 7.83)	0.433
Drink	0.43 (0.05, 3.68)	0.441
SBPhighest before surgery	1.01 (0.98, 1.04)	0.439
DBPhighest before surgery	1.00 (0.98, 1.01)	0.841
Hypertension	1.65 (0.49, 5.60)	0.421
Stroke	1.64 (0.16, 17.13)	0.680
Diabetes	1.56 (0.42, 5.82)	0.512
**elevation of hormone**		
MN	0.99 (0.29, 3.38)	0.987
NMN	1.43 (0.28, 7.27)	0.666
3-MT	3.42 (0.70, 16.63)	0.128
any one of MNs	0.92 (0.17, 4.84)	0.918
all of MNs	11.27 (0.94, 135.24)	**0.056**
**Tumor**		
ectopic tumor	0.70 (0.14, 3.55)	0.666
tumor size	0.87 (0.68, 1.12)	0.290

BMI, body mass index; SBP, systolic blood pressure; DBP, diastolic blood pressure; MN, metanephrine; NMN, normetanephrine; 3-MT, 3-methoxytyramine; MNs, metanephrine and normetanephrines.

Bold values indicate P ≤ 0.1.

Overall, elevation of single hormone (MN, NMN, 3-MT) or any one of MNs was not associated with SMI (P=0.987, 0.666, 0.128 and 0.918, respectively). However, compared with patients with no elevation of MNs, the OR for patients with all MNs elevation was 11.27 (95% CI, 0.94—135.24)(P= 0.056).

Given that age was related to LV GLS (5) (OR: 0.97; 95% CI, 0.93—1.01; P= 0.100) and small sample of the study, multiple regression analysis was performed with age adjusted only (**Table 3**). Elevation of all MNs was associated with SMI (OR: 16.54; 95% CI, 1.22—223.62; P= 0.035), which was in accordance with the results in univariate analysis.

**Table 3:** Results of multivariate analysis.

Variables	OR (95% CI)	P value
Age	0.96 (0.92, 1.00)	0.062
elevation of all MNs	16.54 (1.22, 223.62)	0.035

MNs metanephrine and normetanephrines.

### Repeatability and reproducibility

The intra- and inter- observer intraclass correlation coefficient (ICC) for LV GLS was 0.968 (95%CI: 0.911 -0.989) and 0.982 (95%CI: 0.949 - 0.994), respectively.

## Discussion

As a severe complication of PPGL, CC was a specific cardiomyopathy disease, influencing the cardiac structure and function due to the circulating catecholamine (CA) beyond physical dosage. It was reported that CA had direct effects on the systolic function of LV myocardium and 8-11% of PPGL patients would develop CC (6–8). Mortality rate of CC patients were high at 33% without surgery (9). Perioperative complications would occur in 20-23% CC patients in spite of adequate preoperative preparation. If CC was missed diagnosis or the patient was not fully prepared for the operation, the incidence of perioperative complications and mortality of PPGL surgery would increase to 67-70% and 6-33%, respectively (10).

Symmetric LV hypertrophy was the main pathological characteristic of CC. Myocarditis was found in about 50-60% of PPGL patients at autopsy, with degeneration, contraction band necrosis, inflammatory cells infiltrating and fibrosis during later period. And sarcomere excess contraction, mitochondria swelling and endoplasmic reticulum dilatation could also be observed clearly under electronic microscope, which were entirely different from coagulative necrosis in myocardial infarction (11). Although different from the pathogenic mechanism of ischemic heart disease, it was uncommon to confirm the diagnosis of CC in the early stage and the diagnosis was made by exclusion of other organic heart disease. CC was mainly manifested as high levels of cardiac injury markers and significant decline of LVEF, which indicated clinical myocardial injury (12). Under this condition, the patients had missed the optimal surgery opportunity, bringing about tremendous challenges for the surgeons to prepare for the operation (13). Meune et al. (2) found that subclinical LV systolic dysfunction actually existed in PPGL patients, while whose echocardiogram report were usually normal at the same time. Their study also showed that subclinical LV systolic dysfunction was related to the circulatory failure during the surgery. Unfortunately, there was no well-defined or standard criterion for SMI in their research. Recently, LV GLS was given more attention and played an essential role in SMI (14). Furthermore, LV GLS was regarded as an accurate and sensitive index in early detection of subclinical alterations in LV longitudinal function, which occured before LVEF impairment, myocardial biomarkers elevation and clinical symptoms (15–17). Therefore, the definition of SMI in this study covered the earliest period of cardiac damage which tended to be ignored usually in clinical practice.

We used TOMTEC in LV GLS measurement in the current study and an absolute number of 18% as the cutoff value according to published literature (3–5). LV GLS of 13 (17.11%) patients was less than 18 in our study, but with normal LVEF and myocardial injury biomarkers. This proportion was firstly reported in our study, indicating that some patients with SMI were overlooked during traditional preoperative procedure. The perioperative risks might have increased in such situations.

Although CA was reported to be associated with myocardial injury in PPGL patients in previous research or hypothesis of mechanism, it was still controversial what risk factors increased the probability of injury. In a 15 cases study, there was no correlation between plasma epinephrine or noradrenaline level and LV structural parameters of echocardiography in 6 LV hypertrophy patients (18). However, noradrenaline was thought to be a risk factor of acute cardial complications in the other study (14). One of the most probable reasons was the episodic secretion of epinephrine, noradrenaline or dopamine, making the tests not necessarily reflecting the plasma concentrations of CA on time (19). Furthermore, myocardial injury might be a chronic pathophysiological process, involving the gradual accumulation of CA (20, 21). MNs, intermediate metabolites of CA, were the most accurate index in reflection of tumor condition and the chronic hormone accumulative process. For a consideration of these problems, only patients who tested for all three MNs (MN, NMN and 3-MT) before surgery were enrolled in our study.

In general, CA could cause myocardial injury through chronic and continuous exposure, overexcitation of receptors and desensitization of beta receptors. Large amounts of studies showed that the mechanisms of different CA acting on myocardial injury seemed to be various. Norepinephrine could augment myocardial oxygen demand, cell death and injury and attenuate cardiac performance, thus leading to systolic heart failure and dilated dilated cardiomyopathy (22). The adrenochrome, which was formed through oxidation of epinephrine by tyrosine (23), induced arrhythmias and sudden cardiac death in anesthetized rats (24). Yates et al. (1) found that dopamine-secreting pheochromocytoma might present with hypotension, on account of the effect on β2-adrenoceptors situated on smooth muscles or peripheral arteries. Based on the above, elevation of single MNs (MN, NMN, 3-MT), any one of MNs and all MNs were all analyzed in our study. The results demonstrated that the co-hypersecretion of three MNS was the only factor associated with SMI. And tomor size was not significant associated with SMI, neither was ectopic tumor, which was consistent with the results of several previous studies (8, 25, 26). Co-hypersecretion of three MNs was first described associated with SMI. This may be partly explained by the inclusion criteria, PPGL patients with completely normal LVEF and myocardial injury biomarker, which was quite different from prior studies. It was speculated that the pathogenic mechanism of SMI was different from clinical myocardial injury. As for the type of CA secretion, in most patients with acute myocardial injury, epinephrine secretion was present (14, 27), revealing that the episodical release of epinephrine might cause sudden cardiac decompensation, as opposed to the persistent storage and release of norepinephrine (28, 29). In this study, it was supposed that co-hypersecretion of three types of CA may cause chronic injury to myocardium in some PPGL patients through various receptors and signal pathways, presenting SMI. On the other hand, sharp rise of certain types of CA may directly cause acute myocardial injury, diagnosed as CC. Although the above hypothesis need further study to confirm, for the patients with co-elevation of three plasma MNs, LV GLS measurement was strongly suggested for the early detection of SMI.

Some limitations could not be ignored in the study. Firstly, inevitable bias existed in the retrospective study. Secondly, based on a small sample, LV GLS<18 patients were few in number. Moreover, lack of postoperative follow-up and echocardiography made it impossible to compare the changes of LV GLS. Lastly, qualitative analysis rather than quantitative analysis was used in the elevation of MNs.

## Conclusions

In summary, MNs might be an important factor influencing myocardial function. All MNs elevation might indicate SMI. If all MNs elevated, LV GLS measurement was recommended for PPGL patients to detect SMI in the absence of decrease LVEF or other heart disease in clinical practice.

## Data availability statement

The datasets presented in this article are not readily available because The data that support the findings of this study are available from the corresponding author upon reasonable request after the request is submitted and formally reviewed and approved by the ethics committee of Peking University First Hospital. Requests to access the datasets should be directed to doczhz@aliyun.com.

## Ethics statement

The studies involving human participants were reviewed and approved by the ethics committee of Peking University First Hospital. Written informed consent from the participants’ legal guardian/next of kin was not required to participate in this study in accordance with the national legislation and the institutional requirements.

## Author contributions

ZZ and YiY: conception and design. YY, CC and WH: acquisition of data and critical revision of the manuscript for important intellectual content. YY and CC: analysis and interpretation of data, drafting of the manuscript. ZZ, YZ and YiY: supervision. All authors contributed to the article and approved the submitted version.

## Funding

This work was supported by the National High Level Hospital Clinical Research Funding (Scientific and Technological Achievements Transformation Incubation Guidance Fund Project of Peking University First Hospital) (2022CX08).

## Conflict of interest

The authors declare that the research was conducted in the absence of any commercial or financial relationships that could be construed as a potential conflict of interest.

## Publisher’s note

All claims expressed in this article are solely those of the authors and do not necessarily represent those of their affiliated organizations, or those of the publisher, the editors and the reviewers. Any product that may be evaluated in this article, or claim that may be made by its manufacturer, is not guaranteed or endorsed by the publisher.
